# Supplementary material for: Analysis of an Indian colorectal cancer faecal microbiome collection demonstrates universal colorectal cancer-associated patterns, but closest correlation with other Indian cohorts
Source: BMC Microbiol. 2023 Mar 2;23:52. doi: 10.1186/s12866-023-02805-0 (PMC9979504; doi:10.1186/s12866-023-02805-0)

**Supplementary figures for Bose *et al.*** Analysis of an Indian colorectal cancer faecal microbiome collection demonstrates universal CRC-associated patterns, but closest correlation with other Indian cohorts.

Supplementary figure S1: Number of features per sample called by DADA2 (A) and Shannon index alpha diversity (B) for the current study and the Indian samples from our previous study, Young *et al*.

Supplementary figure S2. Adonis PERMANOVA comparison of the current study and Indian samples from our previous study. R-squared refers to amount of Bray-Curtis variation associated with each metadata category. Status is cancer vs healthy volunteer. Study refers to the current study vs the previous work. P-value is indicated by: *** - p <= 0.001; ** - p <= 0.01; * - p <= 0.05; ‘ – p <= 0.1.

Supplementary figure S3 (next four pages). LEfSe results comparing (A) cancer versus volunteer for merged datasets of Young *et al* with the current study, (B) just the Indian samples of Young *et al* and the current study, (C) just the current study, and (D) metagenomic samples from Gupta *et al.*


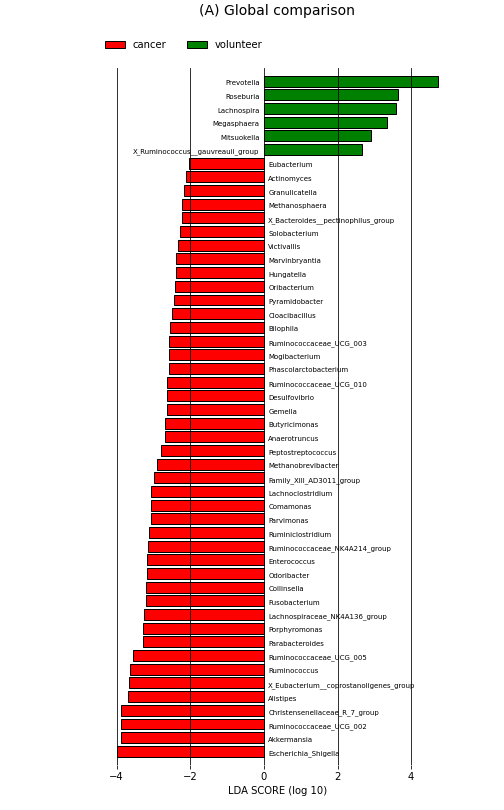


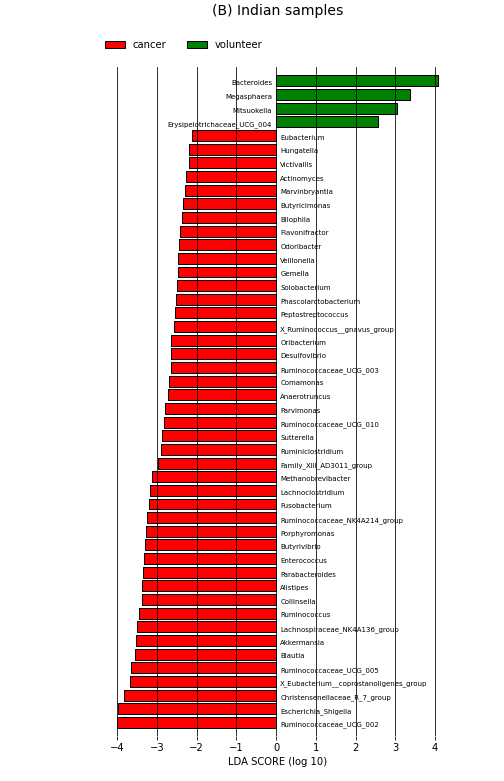


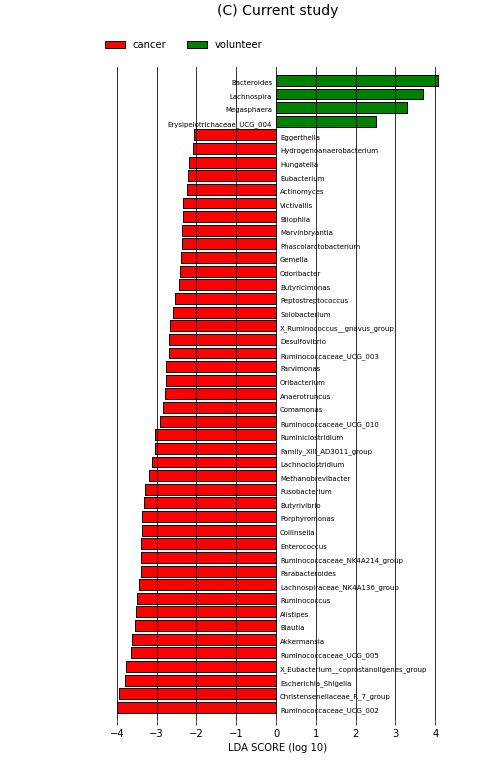


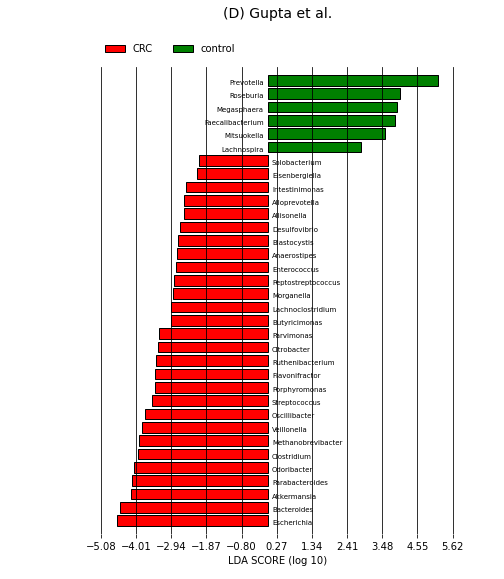

Supplement: Supplementary file 2 — Additional file 2: Supplementary figure S1. Number of features per sample called by DADA2 (A) and Shannon index alpha diversity (B) for the current study and the Indian samples from our previous study, Young et al. Supplementary figure S2. Adonis PERMANOVA comparison of the current study and Indian samples from our previous study. R-squared refers to amount of Bray-Curtis variation associated with each metadata category. Status is cancer vs healthy volunteer. Study refers to the current study vs the previous work. P-value is indicated by: *** - p <= 0.001; ** - p <= 0.01; * - p <= 0.05; ‘ – p <= 0.1. Supplementary figure S3. LEfSe results comparing (A) cancer versus volunteer for merged datasets of Young et al with the current study, (B) just the Indian samples of Young et al and the current study, (C) just the current study, and (D) metagenomic samples from Gupta et al. [file 12866_2023_2805_MOESM2_ESM.docx]
